# Chronic health conditions after childhood Langerhans cell histiocytosis: Results from the Swiss Childhood Cancer Survivor Study

**DOI:** 10.1007/s11764-024-01544-z

**Published:** 2024-02-14

**Authors:** Tomáš Sláma, Luzius Mader, Maša Žarković, Reta Malär, Alexandra Schifferli, Nicolas X. von der Weid, Claudia E. Kuehni, Christina Schindera

**Affiliations:** 1 Childhood Cancer Research Group, Institute of Social and Preventive Medicine, University of Bern, Bern, Switzerland; 2 Graduate School for Cellular and Biomedical Sciences, University of Bern, Bern, Switzerland; 3 Graduate School for Health Sciences, University of Bern, Bern, Switzerland; 4 Department of Paediatrics, Cantonal Hospital Graubuenden, Chur, Switzerland; 5 Division of Paediatric Oncology/Haematology, University Children’s Hospital Basel, University of Basel, Basel, Switzerland; 6 Paediatric Oncology, Inselspital, Bern University Hospital, University of Bern, Bern, Switzerland

**Keywords:** Langerhans cell histiocytosis, Rare disease, Childhood cancer survivors, Cohort study, Chronic health conditions, Late effects

## Abstract

**Purpose:**

Langerhans cell histiocytosis (LCH) is a rare disease characterized by dysregulated proliferation of myeloid marrow progenitors and subsequent organ infiltration. While LCH is associated with a favorable prognosis, some survivors may develop chronic health conditions (CHC) because of the disease. In this study, we aimed to assess the spectrum and prevalence of CHC among LCH survivors compared with siblings and identify factors associated with the development of CHC.

**Methods:**

The Swiss Childhood Cancer Survivor Study sent questionnaires to all ≥ 5-year LCH survivors registered in the Swiss Childhood Cancer Registry and diagnosed between 1976 and 2015. Siblings also received similar questionnaires. We compared CHC prevalence between LCH survivors and siblings and used logistic regression to identify determinants of CHC.

**Results:**

A total of 123 LCH survivors participated in the study, with a response rate of 69%. Median time since diagnosis was 13 years (interquartile range 9–20). Among LCH survivors, 59% had at least one CHC. Cardiovascular (13% vs. 6%), endocrine (15% vs. 2%), musculoskeletal (22% vs. 13%), and digestive (15% vs. 8%) CHC were more common among LCH survivors compared to siblings (all *p* < 0.05). Factors most strongly associated with the occurrence of CHC were multisystem LCH, multifocal bone involvement, and involvement of the pituitary gland.

**Conclusions:**

More than half of long-term LCH survivors suffered from one or more CHC and were affected considerably more than siblings.

**Implications for Cancer Survivors:**

LCH survivors in follow-up care should be screened especially for cardiovascular, endocrine, musculoskeletal, and digestive conditions.

**Supplementary Information:**

The online version contains supplementary material available at 10.1007/s11764-024-01544-z.

## Introduction

Langerhans cell histiocytosis (LCH) is a rare disease characterized by dysregulated proliferation of myeloid marrow progenitors and subsequent organ infiltration caused by somatic mutations in the mitogen-activated protein kinase pathway [1]. Incidence among children younger than 15 years is 4–9 per million per year [2–5]. LCH more often affects males than females with a ratio of 1.2–1.5:1 [2–5]. Clinical manifestations vary and range from spontaneously healing isolated osteolytic lesions to a lymphoma-like syndrome with fatal multiorgan failure [6]. Based on the number of organ systems affected, LCH is classified into single system disease (SS-LCH) and multisystem disease (MS-LCH) [7]. For SS-LCH, treatment options range from a “wait and see” approach, resection, topical steroids, and radiotherapy to systemic treatment with prednisone and vinblastine [8, 9]. For MS-LCH, systemic therapy includes prednisone and vinblastine over 12–24 months possibly escalated with mercaptopurine for patients with risk organ (hematopoiesis, liver, and spleen) involvement or with cytosine arabinosides for refractory disease [9, 10]. Although the 5-year survival rate of LCH without risk organ involvement is close to 100%, it is only 60–80% among MS-LCH patients with risk organ involvement [8, 9]. Since the LCH prognosis is rather favorable, the population of LCH survivors is growing worldwide.

Survivors of childhood cancer are at risk of chronic health conditions (CHC) [11] caused by the disease itself and from chemotherapy and radiotherapy [12]. Diabetes insipidus (DI), orthopedic abnormalities, hearing loss, and neurological consequences were reported as most common CHC among LCH survivors [13, 14]. However, no previous studies compared the prevalence of CHC to healthy peers [13–22]. Previous studies included only survivors of skeletal LCH [22], excluded survivors of single system unifocal LCH [14], and reported about survivors from single centers only [15, 16, 18, 19, 21] or only about survivors who received systemic therapy [14, 17]. Since a comprehensive, population-based description of CHC among LCH survivors is lacking, we describe the spectrum and prevalence of CHC among LCH survivors compared with siblings and describe factors associated with CHC in our study.

## Methods

### Design and study setting

The Swiss Childhood Cancer Survivor Study (SCCSS) is a population-based, long-term follow-up study of all childhood cancer survivors (CCS) diagnosed with cancer between 1976 and 2015 who survived at least 5 years after diagnosis and were registered in the Swiss Childhood Cancer Registry (ChCR) [23]. ChCR centrally registers all children and adolescents diagnosed with leukemia, lymphoma, central nervous system (CNS) tumors, malignant solid tumors, and Langerhans cell histiocytosis before age 21 in Switzerland [24, 25]. Cancer diagnoses in the ChCR were verified by a cytological or histological analysis in 94% of patients [24].

Between 2007 and 2022, all ≥ 5-year survivors received a standardized SCCSS questionnaire which is based on those used in North American and the British childhood cancer survivor studies [26, 27]. For CCS aged 5–15 years, parents were asked to complete the questionnaire [28]. CCS of age ≥ 16 years completed the questionnaire by themselves [29]. We asked survivors for consent to contact their siblings as the comparison group. Siblings received the same questionnaire between 2009 and 2022 without cancer-related questions. For our study, we analyzed questionnaires completed by LCH survivors and siblings of all participating CCS as a control group. Survivors of relapsed or refractory LCH were included.

### Outcome: chronic health conditions

From the questionnaire, we collected information on CHC involving cardiovascular, pulmonary, endocrine, auditory, visual, musculoskeletal, renal, digestive, and neurological systems. For better comparability with other CCS studies [26, 27], and to investigate also conditions which were previously not described in connection with LCH, we included a broad spectrum of conditions. These can be found in Supplemental Table S1. If at least one corresponding CHC was present, we classified survivors as having an affected organ system. If information about health conditions was missing, we assumed conditions were not present, as done previously [29]. For informative purposes, we calculated proportions of missing information about CHC among LCH survivors and siblings.

### Explanatory variables

Questionnaires included information about age at study, sex, migration background, and language region in Switzerland. We obtained the following clinical and treatment-related characteristics from ChCR: age at diagnosis, time since diagnosis, treatment period, and LCH classification (single system and multisystem). ChCR further provided information on involvement of the following organ systems: bone unifocal; bone multifocal; skin; lymph nodes; lung; CNS; pituitary gland; and other organs. We coded treatment modalities (wait and see; surgery; chemotherapy; radiotherapy) and chemotherapeutic agents used (prednisone; vinblastine; mercaptopurine; other drugs) as binary variables. The “wait and see” approach was determined as lack of any treatment. In the case of surgery, chemotherapy, or radiotherapy, combination with another treatment modality is possible. To avoid misinterpretation, we did not include data on LCH reactivations from the ChCR, since these were underreported in our population (8%) compared to other studies (30–36%) [30, 31]. We calculated body mass index (BMI) based on self-reported height and weight. We classified BMI according to the World Health Organization definition [32].

### Statistical analysis

For better comparison of LCH survivors with siblings, we standardized siblings for sex, age at study, migration background, and Swiss language region according to the distribution of survivors [33, 34]. We used multivariable logistic regression with sibling status as outcome to calculate appropriate weights. We set the weight for LCH survivors at 1; we based all subsequent analyses on weighted siblings.

To allow comparisons with previous studies [14, 22], we additionally stratified survivors into two groups: single system bone unifocal disease (SS BU) and other LCH forms combined. We used chi-squared tests to compare CHC prevalence affecting different organ systems between survivors and weighted siblings. We included organ systems significantly more affected (*p* < 0.05; Supplemental Table S1) among survivors than siblings into the logistic regression. We fitted logistic regression models to identify associations between organ-specific CHC and demographic, clinical, and treatment-related explanatory variables. We created separate models for each organ system. Based on previous literature and to avoid overfitting given the low number of LCH survivors, we decided a priori to adjust each model for two potential confounding factors: age at study and sex [35–37]. We performed all analyses using Stata, version 16.1 (College Station, TX, USA).

## Results

### Characteristics of study population

Of 196 eligible LCH survivors, 1 had died and 16 could not be contacted for lack of valid address. We sent 179 remaining survivors questionnaires by post; 123 survivors returned completed questionnaires (response rate 69%; Supplemental Fig. 1). Participating survivors were older than non-participants and more likely treated with surgery (Supplemental Table S2). We identified no other differences between participating and non-participating LCH survivors. Sixty-three percent of survivors were male, median age at study 20 years (interquartile range [IQR] 15–26), and median age at diagnosis 5 years (IQR 2–10; Table 1). Twenty percent of survivors suffered from MS-LCH. Sites most often involved were unifocal bone involvement (56%), multifocal bone involvement (24%), and skin (15%). Forty-six percent of survivors were treated with surgery, 47% with chemotherapy, and 9% with radiotherapy. Prednisone (47%), vinblastine (43%), and mercaptopurine (17%) were chemotherapeutics most often used. Median BMI of participants was 22 (IQR 20–25). The sibling population included 999 participants (Table 1).

**Table 1:** Demographic, clinical, and treatment-related characteristics of LCH survivors and siblings

	Survivors *N*=123 (%)^a^	Siblings (non-weighted) *N*=999 (%)^a^	Siblings (weighted) (%)^a,b^
Sex			
Male	78 (63)	448 (45)	(64)
Female	45 (37)	551 (55)	(36)
Age at study, years, median [IQR]	20 [15–26]	25 [18–32]	-
≤ 15 years	32 (26)	154 (15)	(30)
16–25 years	56 (46)	373 (37)	(43)
≥ 26 years	35 (28)	472 (47)	(27)
Swiss language region			
German	89 (72)	781 (78)	(73)
French or Italian	34 (28)	218 (22)	(27)
Migration background, yes	24 (20)	161 (16)	(20)
Age at diagnosis, years, median [IQR]	5 [2–10]	-	-
0–4 years	56 (46)	-	-
5–9 years	35 (28)	-	-
10–20 years	32 (26)	-	-
Time since diagnosis, years, median [IQR]	13 [9–20]	-	-
5–9 years	39 (32)	-	-
10–19 years	52 (42)	-	-
>20 years	32 (26)	-	-
Treatment period			
1976–1990	36 (29)	-	-
1991–2000	42 (34)	-	-
2001–2015	45 (37)	-	-
Classification			
Single system^c^	99 (80)	-	-
Multisystem	24 (20)	-	-
Involvement			
Bone unifocal	69 (56)	-	-
Bone multifocal	30 (24)	-	-
Skin	19 (15)	-	-
Lymph nodes	7 (6)	-	-
Lung	3 (3)	-	-
CNS	4 (3)	-	-
Pituitary gland	7 (6)	-	-
Other organ^d^	14 (11)	-	-
Treatment			
Wait and see	19 (15)	-	-
Surgery	56 (46)	-	-
Chemotherapy	58 (47)	-	-
Radiotherapy	11 (9)	-	-
Chemotherapeutic agents used			
Prednisone	58 (47)	-	-
Vinblastine	53 (43)	-	-
Mercaptopurine	21 (17)	-	-
Other drug^e^	14 (11)	-	-
Body mass index, kg/m^2^, median [IQR]^f^	22 [20–25]	22 [20–25]	-
Underweight	18 (15)	112 (11)	-
Normal weight	64 (52)	638 (64)	-
Overweight or obese	25 (20)	226 (23)	-
*Missing*	*16 (13)*	*23 (2)*	-

Abbreviations: *N*, number; *IQR*, interquartile range; *CNS*, central nervous system

^a^Column percentage given

^b^Calculated on weighted analysis (weights on age at study, sex, Swiss language region, and migration background according to the distribution among survivors)

^c^Organs affected by single system disease: bone unifocal (*N* = 61), bone multifocal (*N* = 20), skin (*N* = 10), lymph nodes (*N* = 3), CNS (*N* = 2), pituitary gland (*N* = 3)

^d^Other organs involved: gingiva (*N* = 1), mediastinum (*N* = 1), soft tissue (*N* = 9), reticuloendothelial system (*N* = 1), rectum (*N* = 1), bone marrow (*N* = 1)

^e^Other chemotherapeutic drugs used: vincristine (*N* = 2), cyclophosphamide (*N* = 1), doxorubicin (*N* = 1), cytarabine (*N* = 3), tacrolimus (*N* = 1), pimecrolimus (*N* = 1), methotrexate (*N* = 2), etoposide (*N* = 6), indomethacin (*N* = 1), cladribine (*N* = 1)

^f^Body mass index classified as underweight (< 18.5 kg/m^2^), normal weight (≥ 18.5– < 25 kg/m^2^), and overweight or obese (≥ 25 kg/m^2^)[32]

### Prevalence of CHC

**Fig. 1:**
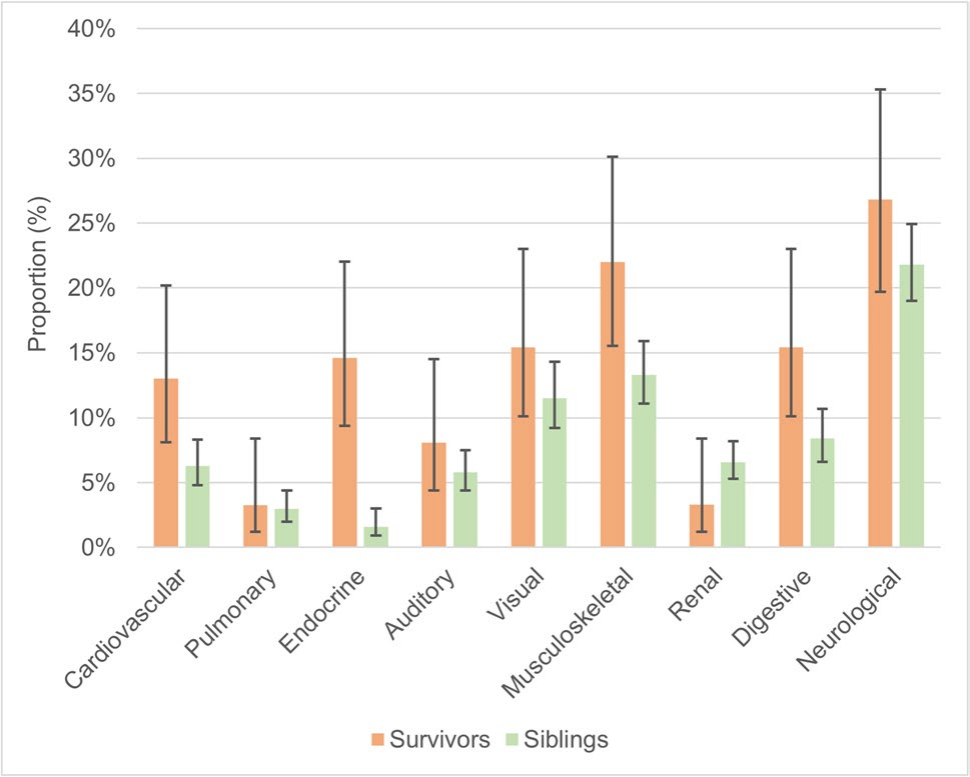
Proportions with 95% confidence intervals of chronic health conditions among LCH survivors compared with weighted siblings. Abbreviations: LCH, Langerhans cell histiocytosis

Fifty-nine percent of participants with LCH had one or more CHC (Supplemental Table S1) and presented more often CHC compared with siblings (*p* = 0.027). Neurological (27%), musculoskeletal (22%), endocrine, visual, and digestive (each 15%) were the most common CHC (Fig. 1). Musculoskeletal (22% vs. 13%), endocrine (15% vs. 2%), digestive (15% vs. 8%), and cardiovascular (13% vs. 6%) CHC were more common among LCH survivors than siblings (*p* < 0.05). We observed neurological CHC in 27% of LCH survivors and 22% of siblings (*p* = 0.202). Among musculoskeletal CHC, prolonged pain in bones or joints (14%) and scoliosis (10%) were most often reported. Common health complications also included diabetes insipidus (11%), growth hormone deficiency (7%), and hypo- or hyperthyroidism (7%) in endocrine CHC, gastro-esophageal reflux disease (10%) and frequent nausea (7%) in digestive CHC, and hypertension (7%) and arrhythmia (5%) in cardiovascular CHC (Supplemental Table S1).

**Fig. 2:**
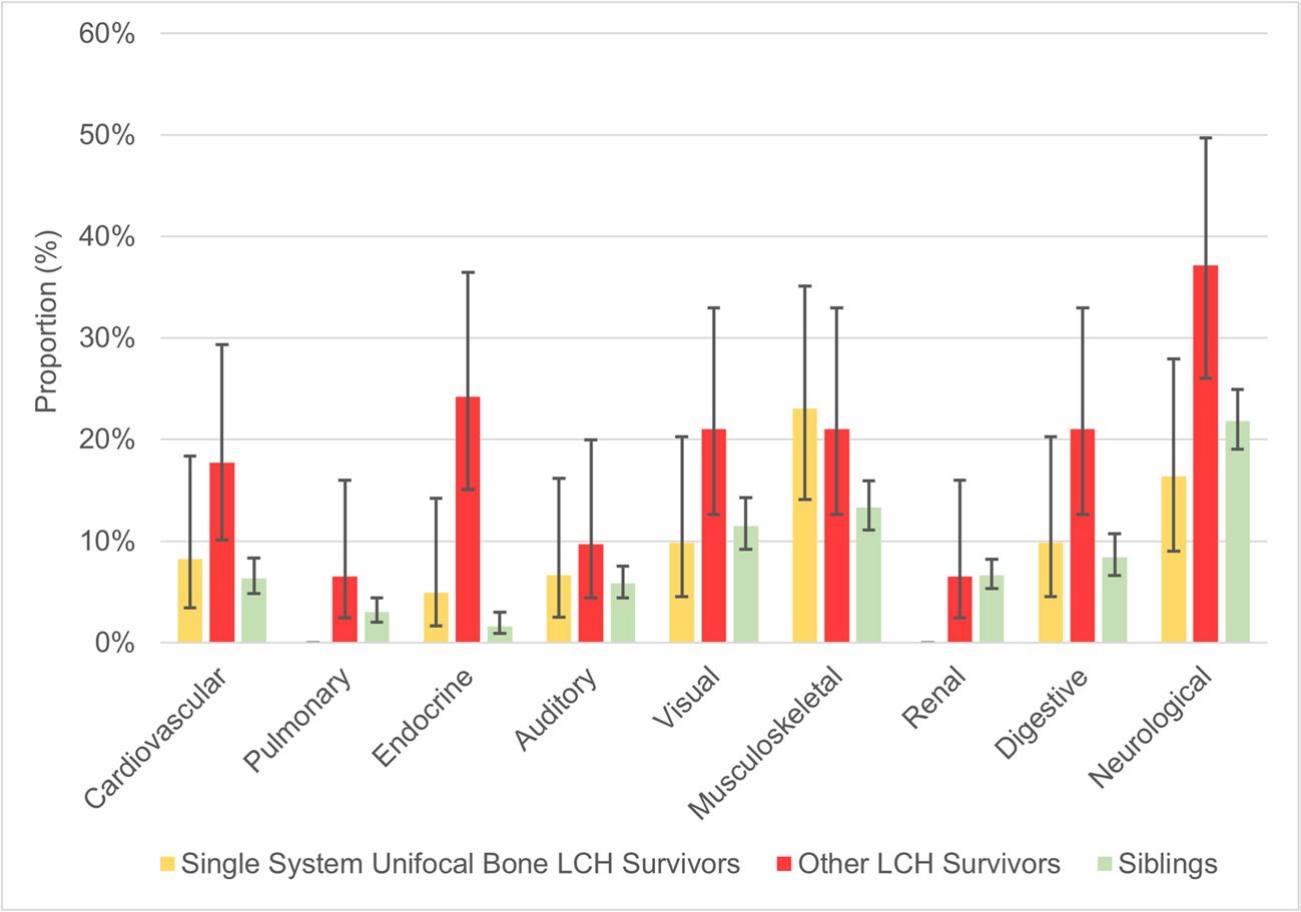
Proportions with 95% confidence intervals of chronic health conditions among survivors of single system unifocal bone LCH and other forms of LCH compared with weighted siblings. Abbreviations: LCH, Langerhans cell histiocytosis

After stratifying LCH survivors into single system unifocal bone and other LCH survivors, we then repeated our analysis. Only musculoskeletal (23% vs. 13%) CHC were more prevalent among single system unifocal bone survivors compared with siblings (Fig. 2; Supplemental Table S3). Among survivors of other LCH forms, neurological (37% vs. 22%), endocrine (24% vs. 2%), visual (21% vs 12%), digestive (21% vs. 8%), and cardiovascular (18% vs. 6%) CHC were more prevalent compared with siblings (all *p* < 0.05). In total, 51% of single system unifocal bone survivors had one or more CHC, while 68% of survivors of other LCH forms had one or more CHC. An overview of the proportions of missing information about CHC among LCH survivors and siblings can be found in Supplemental Table S4.

### Factors associated with CHC among survivors

Endocrine CHC (Table 2) were associated with MS-LCH (OR 3.9; 95% CI 1.3–12.0), multifocal bone involvement (OR 3.4; 95% CI 1.1–10.1), pituitary gland involvement (OR 47.5; 95% CI 5.2–432.7), chemotherapy (OR 5.1; 95% CI 1.5–16.9), and the chemotherapeutic agents prednisone (OR 3.7; 95% CI 1.2–11.5), vinblastine (OR 4.5; 95% CI 1.4–14.2), and mercaptopurine (OR 4.1; 95% CI 1.4–12.6). LCH survivors treated with surgery were less likely to suffer from musculoskeletal CHC (OR 0.3; 95% CI 0.1–0.9). We found that LCH survivors with multifocal bone involvement (OR 4.2; 95% CI 1.4–12.7) treated with mercaptopurine (OR 3.7; 95% CI 1.2–11.7) or those with overweight or obesity (OR 10.4; 95% CI 2.2–48.4) more likely experienced cardiovascular CHC. Digestive CHC were associated with involvement of pituitary gland (OR 12.0; 95% CI 2.2–64.6). LCH survivors with unifocal bone involvement (OR 0.3; 95% CI 0.1–1.0) and treated with surgery (OR 0.3; 95% CI 0.1–0.9) were less likely to experience digestive CHC.

**Table 2:** Determinants of chronic health conditions among LCH survivors from logistic regression models adjusted for sex and age

		Cardiovascular CHC		Endocrine CHC		Musculoskeletal CHC		Digestive CHC	
		OR^a^ (95% CI)	*p*-value^b^	OR^a^ (95% CI)	*p*-value^b^	OR^a^ (95% CI)	*p*-value^b^	OR^a^ (95% CI)	*p*-value^b^
Demographic characteristics									
*Sex*			0.941		0.157		0.175		0.663
	Male	ref		ref		ref		ref	
	Female	1.0 (0.4–3.1)		0.4 (0.1–1.4)		1.8 (0.8–4.4)		1.3 (0.4–3.5)	
*Age at study*			0.203		0.877		0.280		**0.028**
	≤ 15 years	ref		ref		ref		ref	
	16–25 years	6.2 (0.7–55.4)		1.4 (0.2–8.5)		2.6 (0.5–12.9)		9.3 (1.1–81.3)	
	≥ 26 years	15.7 (0.3–725.8)		1.2 (0.0–32.0)		1.6 (0.1–23.2)		n.a	
*Swiss language region*			0.820		0.172		0.943		0.543
	German	ref		ref		ref		ref	
	French or Italian	0.9 (0.3–2.9)		0.4 (0.1–1.6)		1.0 (0.4–2.8)		1.4 (0.5–4.4)	
*Migration background*^*c*^									
	Yes	1.4 (0.4–5.1)	0.604	1.9 (0.6–6.4)	0.304	0.9 (0.3–2.8)	0.901	3.0 (1.0–9.2)	0.055
Clinical and treatment-related characteristics									
*Age at diagnosis*			0.451		0.209		0.349		0.511
	0–4 years	ref		ref		ref		ref	
	5–9 years	0.4 (0.1–1.8)		n.a		0.8 (0.2–2.5)		0.5 (0.1–1.8)	
	10–20 years	0.6 (0.1–2.5)		0.4 (0.1–1.7)		1.8 (0.6–5.6)		0.8 (0.2–2.9)	
*Time since diagnosis*			0.902		0.304		0.106		0.520
	5–9 years	ref		ref		ref		ref	
	10–19 years	0.9 (0.2–3.4)		0.5 (0.1–1.7)		0.5 (0.2–1.4)		1.9 (0.4–7.9)	
	> 20 years	1.3 (0.2–9.0)		1.1 (0.2–7.1)		0.2 (0.0–0.9)		1.0 (0.1–7.1)	
*Treatment period*			0.466		0.784		0.199		0.492
	1976–1990	ref		ref		ref		ref	
	1991–2000	0.7 (0.2–2.8)		0.7 (0.2–2.9)		1.6 (0.5–5.8)		1.3 (0.4–4.6)	
	2001–2015	0.3 (0.0–2.1)		1.0 (0.2–5.6)		3.7 (0.8–17.0)		0.5 (0.1–3.2)	
*Classification*			0.946		**0.019**		0.696		0.108
	Single system	ref		ref		ref		ref	
	Multisystem	1.0 (0.2–3.7)		3.9 (1.3–12.0)		1.2 (0.4–3.7)		2.6 (0.8–8.6)	
*Involvement*^*c*^									
	Bone unifocal	0.3 (0.1–0.9)	**0.023**	0.2 (0.0–0.5)	**0.001**	0.8 (0.3–2.0)	0.656	0.3 (0.1–1.0)	**0.045**
	Bone multifocal	4.2 (1.4–12.7)	**0.013**	3.4 (1.1–10.1)	**0.031**	1.2 (0.4–3.3)	0.722	1.4 (0.4–4.5)	0.585
	Skin	1.4 (0.3–5.8)	0.646	1.9 (0.5–7.0)	0.345	1.1 (0.3–4.0)	0.835	1.6 (0.4–6.5)	0.538
	Pituitary gland	1.1 (0.1–10.3)	0.910	47.5 (5.2–432.7)	** < 0.001**	3.6 (0.7–18.2)	0.133	12.0 (2.2–64.6)	**0.004**
*Treatment*^*c*^									
	Wait and see	1.4 (0.3–5.4)	0.671	0.3 (0.0–2.4)	0.177	2.2 (0.7–6.8)	0.202	1.2 (0.3–5.3)	0.771
	Surgery	0.6 (0.2–1.9)	0.414	0.8 (0.3–2.2)	0.625	0.3 (0.1–0.9)	**0.026**	0.3 (0.1–0.9)	**0.029**
	Chemotherapy	2.2 (0.7–6.5)	0.165	5.1 (1.5–16.9)	**0.004**	1.2 (0.5–3.0)	0.657	2.3 (0.8–6.6)	0.126
	Radiotherapy	0.6 (0.1–5.2)	0.621	0.5 (0.1–4.7)	0.545	0.6 (0.1–3.0)	0.480	n.a	n.a
*Chemotherapeutic agents used*^*c*^									
	Prednisone	2.2 (0.7–6.6)	0.162	3.7 (1.2–11.5)	**0.017**	1.2 (0.5–3.0)	0.652	2.4 (0.8–6.9)	0.110
	Vinblastine	2.7 (0.9–8.5)	0.073	4.5 (1.4–14.2)	**0.007**	0.9 (0.4–2.2)	0.778	1.3 (0.5–3.9)	0.598
	Mercaptopurine	3.7 (1.2–11.7)	**0.033**	4.1 (1.4–12.6)	**0.016**	1.2 (0.4–3.7)	0.757	1.5 (0.4–5.2)	0.540
*Body mass index*			**0.003**		0.324		0.673		0.825
	Normal weight	ref		ref		ref		ref	
	Underweight	n.a		0.4 (0.0–4.0)		1.3 (0.4–4.5)		0.6 (0.1–3.3)	
	Overweight or obese	10.4 (2.2–48.4)		2.2 (0.6–8.1)		0.6 (0.2–2.1)		0.8 (0.2–2.9)	

Abbreviations: *CHC*, chronic health conditions; *OR*, odds ratio; *95% CI*, 95% confidence interval; *n.a.*, not available for lack of observations; *ref*, reference group; *CNS*, central nervous system

^a^OR from adjusted univariable logistic regression models: OR > 1 indicate higher likelihood of existing CHC; OR < 1 indicate lower likelihood of an existing chronic health condition

^b^Global *p*-value calculated from likelihood-ratio test

^c^ “No” used as a reference

## Discussion

In this nationwide and population-based study, we showed that LCH survivors suffered more often from CHC compared with siblings. This was particularly pronounced for musculoskeletal, endocrine, digestive, and cardiovascular CHC. Most prominent factors associated with occurrence of these CHC were MS-LCH, multifocal bone involvement, involvement of pituitary gland, and treatment with chemotherapeutic agents.

In our study, 59% of LCH survivors reported at least one CHC, which aligns with findings of previous studies. Chow et al. reported 56% of 70 LCH survivors suffered from CHC in a single-center study of retrospectively reviewed medical records after a median follow-up time of 10 years [21]. Similarly, Willis et al. reported 64% of 51 LCH survivors with median time since diagnosis of 8 years with CHC in a single-center study [18]. Ceci et al. reported 48% of 90 LCH survivors with CHC in their prospective multicenter study of < 5-year LCH survivors [17]. In contrast, a Japanese study reported only 34% of 317 LCH survivors with multifocal bone involvement or MS-LCH treated with cytarabine-based protocols presented with CHC at a median follow-up of 12 years [14]. Differences in inclusion criteria and CHC assessment may explain varying frequencies of CHC.

The spectrum of CHC after LCH differs based on LCH form. While survivors of single system unifocal bone LCH suffer more often from musculoskeletal CHC only, survivors of other LCH forms may experience neurological, endocrine, visual, digestive, and cardiovascular CHC—important information for clinicians to provide better-tailored follow-up care. Neurological, endocrine (especially diabetes insipidus), and musculoskeletal CHC were reported previously as most prevalent CHC among LCH survivors, which corresponds with our findings [13, 14, 21]. Cardiovascular CHC have not been described in the context of LCH so far while digestive and visual only rarely [13, 14]. For cardiovascular CHC, we saw a strong association with overweight—an established cardiovascular risk factor. Since digestive CHC are most strongly associated with pituitary gland involvement, the cause of these CHC might be the use of desmopressin in patients with diabetes insipidus. The most common side effects of desmopressin are digestive conditions, such as nausea, diarrhea, and abdominal pain [38]. Higher prevalence of visual CHC among survivors of other LCH forms could be caused by the therapeutical use of prednisone in MS-LCH which has been associated with ophthalmological late effects [39].

High prevalence (37%) of neurological CHC among survivors of other LCH forms (i.e., mainly MS-LCH) is remarkable. Weakness or inability to move arms or legs, balance disorders, and dysphagia or chewing difficulties—conditions more often reported among survivors of other LCH forms in our study—could be compatible with neurodegenerative CNS-LCH (ND-CNS-LCH) disease [40]. ND-CNS-LCH is one of the most devastating, yet rare consequences of LCH characterized clinically by progressive cerebellar ataxia, cognitive disorders, and impairment of cranial nerves [41] and radiologically by magnetic resonance imaging signal changes in cerebellum, basal ganglia, or pons [42]. CD8 + lymphocytes infiltrating the brain of affected individuals are a possible pathophysiological explanation of this disease [42]. Diagnosis of this poorly understood condition is challenging and often missed, and comprises imaging and neurocognitive assessments [43, 44]. It is unclear how many of our participants have been diagnosed with ND-CNS-LCH, but some might have been affected.

MS-LCH is a known risk factor for developing CHC in LCH survivors [13, 22]. In our study, MS-LCH was particularly associated with endocrine CHC, which is probably from the frequent involvement of the pituitary gland in MS-LCH causing diabetes insipidus, hypogonadism, and growth hormone deficiency. Our study also showed multifocal bone involvement was associated with CHC, especially cardiovascular and endocrine CHC. Some survivors with multifocal bone involvement may have had concurrent involvement of the pituitary gland (as part of their MS-LCH), which could explain the association between multifocal bone involvement and endocrine CHC. Chemotherapy and individual chemotherapeutic agents also showed an association with endocrine CHC. Neither prednisone nor vinblastine or mercaptopurine is known as causing endocrine CHC. Therefore, we interpreted the association of chemotherapy also as a proxy for MS-LCH.

Our study is a nationwide, population-based study on the prevalence and factors associated with the development of CHC among LCH survivors. We included involvement of all organs, all clinical manifestations of LCH, and all treatment regimens applied nationally. Previous studies were often single-center studies, included MS-LCH only, or certain treatment regimens [14, 16, 18]. Importantly, our study included siblings as the healthy comparison group. Our high 69% response rate supports the representativeness of our study population. We previously showed non-response bias plays only a minor role in SCCSS [45], which is supported by our comparison of participating and non-participating LCH survivors. We cover all treatment periods from 1976 to 2015. Another strength of our study lies in the high-quality clinical and treatment-related data provided by ChCR. However, using self-reported data on health conditions can also introduce reporting bias since certain conditions, such as mild hearing loss, often remain unnoticed unless clinically tested [46]. Given LCH rarity, our numbers of survivors with certain clinical characteristic were relatively small and our study possibly lacks statistical power in certain subgroup analyses. As a result, some associations between CHC and survivor characteristics, such as the identified association between multifocal bone LCH and cardiovascular CHC, might have occurred by chance and need to be confirmed in other populations. Additionally, the long duration of study enrollment with eligibility since 1976 means that LCH survivors from very different treatment eras are included and put together. Therefore, findings regarding CHC in our sample are representative for LCH patients treated in the past, but may not be generalizable (or necessarily relevant) to those treated nowadays with novel treatment modalities such as BRAF or MEK inhibitors.

Another limitation of our study is that all CHC of study participants were captured in our analysis, irrespective whether they occurred first before or after LCH diagnosis. However, as we use siblings as control group, we get an estimation of the CHC burden independent of LCH diagnosis. Also, we did not stratify CHC according to severity. Therefore, mild and severe CHC are equated, which is a further limitation of our study.

We showed more than half of long-term LCH survivors suffered from at least one chronic health condition and were affected considerably more than siblings. Clinicians in pediatric cancer survivorship programs should be vigilant regarding musculoskeletal, endocrine, neurological, digestive, and cardiovascular CHC in multisystem LCH, multifocal bone disease survivors, and those with pituitary gland involvement. LCH survivors with suspected neurological symptoms or deficits should be referred early to a neurologist for further evaluation including neuropsychological assessment and imaging studies.

## Supplementary Information

Below is the link to the electronic supplementary material.

Supplementary file1 (DOCX 108 KB)

## Data Availability

Researchers interested in collaborative work can contact the corresponding author (Christina Schindera; christina.schindera@unibe.ch) to discuss planned projects or analyses of existing data.
