# Supplementary material for: Chronic health conditions after childhood Langerhans cell histiocytosis: Results from the Swiss Childhood Cancer Survivor Study
Source: J Cancer Surviv. 2024 Feb 14;19(4):1212–21. doi: 10.1007/s11764-024-01544-z (PMC12283886; doi:10.1007/s11764-024-01544-z)
Supplement: Supplementary file 1 — Supplementary file1 (DOCX 108 KB) [file 11764_2024_1544_MOESM1_ESM.docx]

SUPPLEMENTAL TABLE S1 Chronic health conditions among Langerhans cell histiocytosis survivors compared with siblings

| **Affected system** | **Symptoms / diseases** | Survivors  N=123 (%) *^a,b^* | Siblings (weighted)  (%) *^a,b,c^* | p^d^ |
| --- | --- | --- | --- | --- |
| Cardiovascular | Arrhythmia^e^ | 6 (5) | (2) |  |
|  | Hypertension | 8 (7) | (3) |  |
|  | Heart failure | 1 (1) | (0) |  |
|  | DVT and pulmonary embolism | 1 (1) | (0) |  |
|  | Angina pectoris | 3 (2) | (1) |  |
|  | Valvular heart disease | 0 (0) | (1) |  |
|  | Stroke | 1 (1) | (0) |  |
|  | *Any cardiovascular CHC* | *16 (13)* | *(6)* | ***0.009*** |
| Pulmonary | Chronic cough^f^ | 3 (2) | (3) |  |
|  | Lung fibrosis | 2 (2) | (0) |  |
|  | Chest wall abnormalities | 1 (1) | (0) |  |
|  | *Any pulmonary CHC* | *4 (3)* | *(3)* | *0.860* |
| Endocrine | Diabetes mellitus^g^ | 0 (0) | (1) |  |
|  | Diabetes insipidus | 13 (11) | (0) |  |
|  | Hypo- or hyperthyroidism | 8 (7) | (1) |  |
|  | Thyroid nodules or tumour | 1 (1) | (0) |  |
|  | Growth hormone deficiency | 9 (7) | (0) |  |
|  | Hypogonadism | 2 (2) | (0) |  |
|  | *Any endocrine CHC* | *18 (15)* | *(2)* | ***<0.001*** |
| Auditory | Mild, moderate, severe hearing loss | 8 (7) | (3) |  |
|  | Tinnitus | 3 (2) | (4) |  |
|  | *Any hearing CHC* | *10 (8)* | *(6)* | *0.308* |
| Visual | Severe visual impairment or blindness^h^ | 3 (2) | (2) |  |
|  | Mild or moderate visual impairment | 11 (9) | (5) |  |
|  | Cataract | 1 (1) | (0) |  |
|  | Dry eye syndrome | 2 (2) | (5) |  |
|  | Eye movement disorders (incl. strabismus) | 4 (3) | (1) |  |
|  | *Any visual CHC* | *19 (15)* | *(12)* | *0.224* |
| Musculoskeletal | Shortened extremities | 4 (3) | (1) |  |
|  | Reduced flexibility of joints | 6 (5) | (4) |  |
|  | Prolonged pain in bones or joints | 17 (14) | (7) |  |
|  | Scoliosis | 12 (10) | (4) |  |
|  | Osteoporosis | 5 (4) | (0) |  |
|  | *Any musculoskeletal CHC* | *27 (22)* | *(13)* | ***0.012*** |
| Renal | Repeated cystitis | 4 (3) | (6) |  |
|  | Repeated nephritis | 2 (2) | (1) |  |
|  | *Any renal CHC* | *4 (3)* | (7) | *0.144* |
| Digestive | Chronic constipation or diarrhoea | 7 (6) | (3) |  |
|  | Gastro-oesophageal reflux disease | 12 (10) | (4) |  |
|  | Problems with oesophagus | 2 (2) | (0) |  |
|  | Frequent nausea^i^ | 9 (7) | (1) |  |
|  | *Any digestive CHC* | *19 (15)* | *(8)* | ***0.015*** |
| Neurological | Weakness or inability to move arms or legs | 11 (9) | (4) |  |
|  | Hypoesthesia | 7 (6) | (2) |  |
|  | Balance disorders | 9 (7) | (2) |  |
|  | Dysphagia or chewing difficulties | 4 (3) | (1) |  |
|  | Anosmia or ageusia | 1 (1) | (1) |  |
|  | Speech disorders^j^ | 3 (2) | (3) |  |
|  | Epilepsy^k^ | 5 (4) | (2) |  |
|  | Migraine | 17 (14) | (12) |  |
|  | *Any neurological CHC* | *33 (27)* | *(22)* | *0.220* |
| *Any CHC* | *None* | *50 (41)* | *(52)* | ***0.027*** |
|  | *One and more* | *73 (59)* | *(48)* |  |

Abbreviations: N, number; DVT, deep venous thrombosis; CHC, chronic health conditions

^a^ Column percentage for total survivor or sibling populations given

^b^ Missing information coded as absence of respective conditions

^c^ Siblings weighted for sex, age at study, migration background, and Swiss language region

^d^ p-value retrieved from chi-square tests comparing survivors and weighted siblings

^e^ Requiring follow-up by a physician

^f^ For more than 3 months

^g^ Controlled with diet or medication

^h^ Unilateral or bilateral

^i^ Without a clear cause

^j^ Including stammering or stuttering

^k^ Including convulsions and blackouts

SUPPLEMENTAL TABLE S2 Comparison of available demographic, clinical, and treatment-related characteristics of participating and non-participating Langerhans cell histiocytosis survivors

|  | Participants  N=123 (%) *^a^* | Non-participants  N=56 (%) *^a^* | p^b^ |
| --- | --- | --- | --- |
| **Demographic characteristics**  Sex  Male  Female  Age at study, years, median [IQR]  Age at study, years, categories  ≤ 15 years  16–25 years  ≥ 26 years  Swiss language region  German  French or Italian  Migration background, yes | 78 (63)  45 (37)  20 [15–26]  32 (26)  56 (46)  35 (28)  89 (72)  34 (28)  24 (20) | 30 (54)  26 (46)  17 [13–21]  24 (43)  24 (43)  8 (14)  36 (64)  20 (36)  16 (29) | 0.250  **0.013**  **0.035**  0.295  0.182 |
| **Clinical and treatment-related characteristics**  Age at diagnosis, years, median [IQR]  Age at diagnosis, years, categories  0–4 years  5–9 years  10–20 years  Time since diagnosis, years, median [IQR]  Time since diagnosis, years, categories  5–9 years  10–19 years  >20 years  Treatment period  1976–1990  1991–2000  2001–2015  Classification  Single system  Multisystem  Involvement  Bone unifocal  Bone multifocal  Skin  Lymph nodes  Lung  CNS  Pituitary gland  Other organ  Treatment  Wait and see  Surgery  Chemotherapy  Radiotherapy | 5 [2–10]  56 (46)  35 (28)  32 (26)  13 [9–20]  39 (32)  52 (42)  32 (26)  36 (29)  42 (34)  45 (37)  99 (80)  24 (20)  69 (56)  30 (24)  19 (15)  7 (6)  3 (3)  4 (3)  7 (6)  14 (11)  19 (15)  56 (46)  58 (47)  11 (9) | 4 [1–9]  30 (54)  13 (23)  13 (23)  10 [8–17]  24 (43)  24 (43)  8 (14)  11 (20)  17 (30)  28 (50)  43 (77)  13 (23)  26 (46)  18 (32)  10 (18)  2 (4)  1 (2)  0 (0)  4 (7)  9 (16)  11 (20)  15 (27)  33 (59)  3 (5) | 0.149  0.635  0.068  0.154  0.207  0.558  0.260  0.281  0.668  0.722  1.000  0.311  0.742  0.470  0.520  **0.021**  0.151  0.554 |

Abbreviations: N, number; IQR, interquartile range; CNS, central nervous system

^a^ Column percentage given

^b^ p-values retrieved from Fischer’s exact test for categorical variables and Student’s t-test for continuous variables

SUPPLEMENTAL TABLE S3 Chronic health conditions among survivors of single system bone unifocal (SS BU) and other forms of Langerhans cell histiocytosis compared with weighted siblings

| **Affected system** | **Symptoms / diseases** | SS BU Survivors  N=61 (%) *^a,b^* | Other LCH Survivors  N=62 (%) *^a,b^* | Siblings (weighted)  (%) *^a,b,c^* | p^d^ | p^e^ |
| --- | --- | --- | --- | --- | --- | --- |
| Cardiovascular | Arrhythmia^f^ | 2 (3) | 4 (6) | (2) |  |  |
|  | Hypertension | 4 (7) | 4 (6) | (3) |  |  |
|  | Heart failure | 0 (0) | 1 (2) | (0) |  |  |
|  | DVT and pulmonary embolism | 0 (0) | 1 (2) | (0) |  |  |
|  | Angina pectoris | 0 (0) | 3 (5) | (1) |  |  |
|  | Valvular heart disease | 0 (0) | 0 (0) | (1) |  |  |
|  | Stroke | 0 (0) | 1 (2) | (0) |  |  |
|  | *Any cardiovascular CHC* | *5 (8)* | *11 (18)* | *(6)* | *0.565* | ***0.001*** |
| Pulmonary | Chronic cough^g^ | 0 (0) | 3 (5) | (3) |  |  |
|  | Lung fibrosis | 0 (0) | 2 (3) | (0) |  |  |
|  | Chest wall abnormalities | 0 (0) | 1 (2) | (0) |  |  |
|  | *Any pulmonary CHC* | *0 (0)* | *4 (6)* | *(3)* | ***<0.001*** | *0.133* |
| Endocrine | Diabetes mellitus^h^ | 0 (0) | 0 (0) | (1) |  |  |
|  | Diabetes insipidus | 1 (2) | 12 (19) | (0) |  |  |
|  | Hypo- or hyperthyroidism | 3 (5) | 5 (8) | (1) |  |  |
|  | Thyroid nodules or tumour | 0 (0) | 1 (2) | (0) |  |  |
|  | Growth hormone deficiency | 1 (2) | 8 (13) | (0) |  |  |
|  | Hypogonadism | 0 (0) | 2 (3) | (0) |  |  |
|  | *Any endocrine CHC* | *3 (5)* | *15 (24)* | *(2)* | *0.076* | ***<0.001*** |
| Auditory | Mild, moderate, severe hearing loss | 3 (5) | 5 (8) | (3) |  |  |
|  | Tinnitus | 1 (2) | 2 (3) | (4) |  |  |
|  | *Any hearing CHC* | *4 (7)* | *6 (10)* | *(6)* | *0.804* | *0.215* |
| Visual | Severe visual impairment or blindness^i^ | 2 (3) | 1 (2) | (2) |  |  |
|  | Mild or moderate visual impairment | 3 (5) | 8 (13) | (5) |  |  |
|  | Cataract | 0 (0) | 1 (2) | (0) |  |  |
|  | Dry eye syndrome | 0 (0) | 2 (3) | (5) |  |  |
|  | Eye movement disorders (incl. strabismus) | 1 (2) | 3 (5) | (1) |  |  |
|  | *Any visual CHC* | *6 (10)* | *13 (21)* | *(12)* | *0.692* | ***0.032*** |
| Musculoskeletal | Shortened extremities | 2 (3) | 2 (3) | (1) |  |  |
|  | Reduced flexibility of joints | 2 (3) | 4 (6) | (4) |  |  |
|  | Prolonged pain in bones or joints | 9 (15) | 8 (13) | (7) |  |  |
|  | Scoliosis | 5 (8) | 7 (11) | (4) |  |  |
|  | Osteoporosis | 0 (0) | 5 (8) | (0) |  |  |
|  | *Any musculoskeletal CHC* | *14 (23)* | *13 (21)* | *(13)* | ***0.039*** | *0.097* |
| Renal | Repeated cystitis | 0 (0) | 4 (6) | (6) |  |  |
|  | Repeated nephritis | 0 (0) | 2 (3) | (1) |  |  |
|  | *Any renal CHC* | *0 (0)* | *4 (6)* | (7) | ***<0.001*** | *0.957* |
| Digestive | Chronic constipation or diarrhoea | 3 (5) | 4 (6) | (3) |  |  |
|  | Gastro-oesophageal reflux disease | 3 (5) | 9 (15) | (4) |  |  |
|  | Problems with oesophagus | 0 (0) | 2 (3) | (0) |  |  |
|  | Frequent nausea^j^ | 2 (3) | 7 (11) | (1) |  |  |
|  | *Any digestive CHC* | *6 (10)* | *13 (21)* | *(8)* | *0.708* | ***0.001*** |
| Neurological | Weakness or inability to move arms or legs | 3 (5) | 8 (13) | (4) |  |  |
|  | Hypoesthesia | 2 (3) | 5 (8) | (2) |  |  |
|  | Balance disorders | 2 (3) | 7 (11) | (2) |  |  |
|  | Dysphagia or chewing difficulties | 0 (0) | 4 (6) | (1) |  |  |
|  | Anosmia or ageusia | 0 (0) | 1 (2) | (1) |  |  |
|  | Speech disorders^k^ | 2 (3) | 1 (2) | (3) |  |  |
|  | Epilepsy^l^ | 1 (2) | 4 (6) | (2) |  |  |
|  | Migraine | 6 (10) | 11 (18) | (12) |  |  |
|  | *Any neurological CHC* | *10 (16)* | *23 (37)* | *(22)* | *0.321* | ***0.007*** |
| *Any CHC* | *None* | *30 (49)* | *20 (32)* | *(52)* | *0.722* | ***0.004*** |
|  | *One and more* | *31 (51)* | *42 (68)* | *(48)* |  |  |

Abbr.: LCH, Langerhans cell histiocytosis; SS BU, single system bone unifocal; N, number; DVT, deep venous thrombosis; CHC, chronic health conditions

^a^Column percentages are given.

^b^Missing information were coded as absence of respective conditions.

^c^Siblings were weighted for sex, age at study, migration background, and Swiss language region.

^d^p-value retrieved from chi-square test comparing survivors of SS BU LCH and weighted siblings.

^e^p-value retrieved from chi-square test comparing survivors of other LCH forms and weighted siblings.

^f^Requiring follow-up by a physician.

^g^For more than 3 months.

^h^Controlled with diet or medication.

^i^Unilateral or bilateral.

^j^Without a clear cause.

^k^Including stammering or stuttering.

^l^Including convulsions and blackouts.

SUPPLEMENTAL TABLE S4 Percentages of missing information about chronic health conditions among survivors of Langerhans cell histiocytosis and siblings

| **Affected system** | **Symptoms / diseases** | Survivors  % ^a^ | Siblings  % ^a^ |
| --- | --- | --- | --- |
| Cardiovascular | Arrhythmia^b^ | 8% | 2% |
|  | Hypertension | 8% | 1% |
|  | Heart failure | 9% | 2% |
|  | DVT and pulmonary embolism | 22% | 7% |
|  | Angina pectoris | 57% | 33% |
|  | Valvular heart disease | 20% | 7% |
|  | Stroke | 11% | 2% |
| Pulmonary | Chronic cough^c^ | 11% | 5% |
|  | Lung fibrosis | 35% | 17% |
|  | Chest wall abnormalities | 9% | 2% |
| Endocrine | Diabetes mellitus^d^ | 10% | 1% |
|  | Diabetes insipidus | 8% | 2% |
|  | Hypo- or hyperthyroidism | 10% | 1% |
|  | Thyroid nodules or tumour | 43% | 0% |
|  | Growth hormone deficiency | 9% | 2% |
|  | Hypogonadism | 16% | 4% |
| Auditory | Mild, moderate, severe hearing loss | 11% | 2% |
|  | Tinnitus | 58% | 48% |
| Visual | Severe visual impairment or blindness^e^ | 19% | 6% |
|  | Mild or moderate visual impairment | 19% | 6% |
|  | Cataract | 11% | 6% |
|  | Dry eye syndrome | 11% | 5% |
|  | Eye movement disorders (incl. strabismus) | 10% | 5% |
| Musculoskeletal | Shortened extremities | 7% | 3% |
|  | Reduced flexibility of joints | 8% | 2% |
|  | Prolonged pain in bones or joints | 8% | 2% |
|  | Scoliosis | 7% | 3% |
|  | Osteoporosis | 35% | 0% |
| Renal | Repeated cystitis | 10% | 2% |
|  | Repeated nephritis | 11% | 2% |
| Digestive | Chronic constipation or diarrhoea | 8% | 2% |
|  | Gastro-oesophageal reflux disease | 34% | 0% |
|  | Problems with oesophagus | 8% | 2% |
|  | Frequent nausea^f^ | 42% | 0% |
| Neurological | Weakness or inability to move arms or legs | 15% | 5% |
|  | Hypoesthesia | 15% | 5% |
|  | Balance disorders | 9% | 15% |
|  | Dysphagia or chewing difficulties | 7% | 2% |
|  | Anosmia or ageusia | 7% | 3% |
|  | Speech disorders^g^ | 15% | 3% |
|  | Epilepsy^i^ | 9% | 3% |
|  | Migraine | 11% | 3% |

Abbreviations: N, number; DVT, deep venous thrombosis; CHC, chronic health conditions

^a^ Column percentage for total survivor or sibling populations given

^b^ Requiring follow-up by a physician

^c^ For more than 3 months

^d^ Controlled with diet or medication

^e^ Unilateral or bilateral

^f^ Without a clear cause

^g^ Including stammering or stuttering

^h^ Including convulsions and blackouts


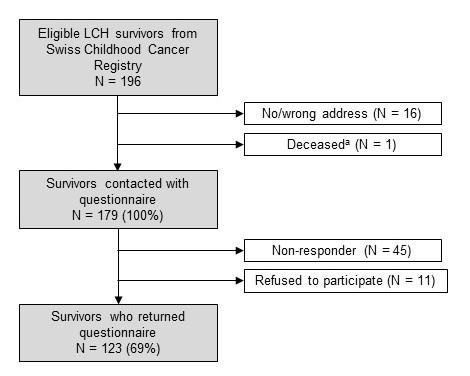


SUPPLEMENTAL FIGURE 1 Flowchart of eligible, contacted, and participating LCH survivors

Abbreviations: LCH, Langerhans cell histiocytosis

^a^ Deceased from causes unrelated to LCH
